# Glutathione Induces Keap1 S-Glutathionylation and Mitigates Oscillating Glucose-Induced β-Cell Dysfunction by Activating Nrf2

**DOI:** 10.3390/antiox13040400

**Published:** 2024-03-27

**Authors:** Xiufang Chen, Qian Zhou, Huamin Chen, Juan Bai, Ruike An, Keyi Zhang, Xinyue Zhang, Hui An, Jitai Zhang, Yongyu Wang, Ming Li

**Affiliations:** 1 Department of Biochemistry and Molecular Biology, School of Basic Medical Sciences, Wenzhou Medical University, Wenzhou 325035, China; 17778912672@163.com (Q.Z.); 2662548575@wmu.edu.cn (H.C.); 17757728680@163.com (J.B.); ark17835207163@wmu.edu.cn (R.A.); zky15957651455@wmu.edu.cn (K.Z.); 2 Cardiac Regeneration Research Institute, School of Basic Medical Sciences, Wenzhou Medical University, Wenzhou 325035, China; qhdzxy@wmu.edu.cn (X.Z.); anhui@wmu.edu.cn (H.A.); jtaizhang@wmu.edu.cn (J.Z.); 3 Institute of Hypoxia Medicine, School of Basic Medical Sciences, Wenzhou Medical University, Wenzhou 325035, China; yongyuwang@wmu.edu.cn

**Keywords:** prediabetes, glucotoxicity, β-cell, glutathione, S-glutathionylation, Keap1, Nrf2

## Abstract

Glutathione (GSH), a robust endogenous antioxidant, actively participates in the modulation of the redox status of cysteine residues in proteins. Previous studies have indicated that GSH can prevent β-cell failure and prediabetes caused by chronic oscillating glucose (OsG) administration. However, the precise mechanism underlying the protective effect is not well understood. Our current research reveals that GSH is capable of reversing the reduction in Nrf2 levels, as well as downstream genes Grx1 and HO-1, in the islet β-cells of rats induced by chronic OsG. In vitro experiments have further demonstrated that GSH can prevent β-cell dedifferentiation, apoptosis, and impaired insulin secretion caused by OsG. Additionally, GSH facilitates the translocation of Nrf2 into the nucleus, resulting in an upregulation of Nrf2-targeted genes such as GCLC, Grx1, HO-1, and NQO1. Notably, when the Nrf2 inhibitor ML385 is employed, the effects of GSH on OsG-treated β-cells are abrogated. Moreover, GSH enhances the S-glutathionylation of Keap1 at Cys273 and Cys288, but not Cys151, in OsG-treated β-cells, leading to the dissociation of Nrf2 from Keap1 and facilitating Nrf2 nuclear translocation. In conclusion, the protective role of GSH against OsG-induced β-cell failure can be partially attributed to its capacity to enhance Keap1 S-glutathionylation, thereby activating the Nrf2 signaling pathway. These findings provide novel insights into the prevention and treatment of β-cell failure in the context of prediabetes/diabetes, highlighting the potential of GSH.

## 1. Introduction

The prevalence of prediabetes has been attributed to the consumption of indulgent food, excessive eating, and insufficient physical activity in modern societies [1,2]. Prediabetes is characterized by the coexistence of insulin resistance and β-cell dysfunction, which occur prior to the detectable changes in glucose levels. Lifestyle modification is considered the fundamental approach for preventing prediabetes, supported by evidence showing a 40–70% reduction in relative risk [2,3,4]. Without effective preventative measures, an annual progression rate of 5–10% from prediabetes to diabetes is observed [3]. The underlying pathogenesis of prediabetes is not yet fully elucidated, but potential factors may include oxidative stress, dysfunction of pancreatic β-cells, insulin resistance, chronic inflammatory responses, and alterations in gut microbiota [1,2,4]. Hence, gaining a thorough understanding of the fundamental mechanisms that contribute to metabolic syndrome induced by dietary intake, particularly the rise in the consumption of high-calorie, low-fiber fast food, is of utmost importance for effective prevention of prediabetes.

Our previous research conducted on rats has demonstrated that oral intake of long-time oscillating glucose (LOsG) for 38 days, four times a day, induces prediabetes. This is evidenced by hypoinsulinemia, glucose intolerance, increased accumulation of reactive oxygen species (ROS), and impaired expression of insulin in β-cells. However, simultaneous administration of glutathione (GSH) totally prevented LOsG/ROS-mediated dysfunction of β-cells and the development of prediabetes [5,6]. This coincides with findings from clinical research indicating that individuals with type 2 diabetes (T2D) exhibit a decreased level of GSH in red blood cells [7,8]. Nonetheless, additional investigations are required to fully understand the protective mechanism of GSH on β-cells.

Pancreatic β-cells exhibit increased susceptibility to chemical compounds that disrupt redox reactions. This heightened sensitivity can be attributed to the fact that these cells depend on ROS as cellular signals for differentiation and insulin secretion stimulation [9,10]. It is well-established that oxidative stress plays a crucial role in β-cell glucotoxicity, particularly in the hyperglycemic environment associated with diabetes [11,12,13]. In addition, due to their low expression of crucial antioxidant enzymes such as catalase and glutathione peroxidase 1, pancreatic β-cells are exceptionally vulnerable and responsive to oxidative stress [14,15]. The decline in β-cell mass and impaired function are key contributing factors in the pathogenesis of T2D [16]. At the onset of T2D, it is estimated that β-cell mass and function decrease by 25–60% [17,18].

GSH, a cellular metabolite that contains thiol groups, plays multiple roles in the formation of iron-sulfur proteins and the elimination of various electrophilic metabolites [19]. Furthermore, GSH serves as a crucial antioxidant molecule utilized by several enzymes to counteract reactive oxygen species (ROS) and regulates the redox state of cysteine residues in proteins [19,20]. In many reactions, reduced GSH is oxidized to form glutathione disulfide (GSSG), which can be converted back to GSH through NADPH-dependent glutathione reductase [20,21]. Protein S-glutathionylation (PSSG) is a specific post-translational modification that involves the conjugation of GSH to the reactive thiol groups of protein cysteines [22]. S-glutathionylation often induces a conformational change that alters the function of the protein or enzyme activity, serving as a novel redox switch or regulator [23,24]. This process occurs under physiological conditions and is promoted in response to oxidative or nitrosative stress [24,25]. Kelch-like ECH-associated protein 1 (Keap1), a redox-sensitive protein rich in cysteine thiols and containing several highly reactive cysteines, can undergo S-glutathionylation. The S-glutathionylation of Keap1 has been reported to activate nuclear factor erythroid 2-related factor 2 (Nrf2) and protect neurons against MPTP-induced oxidative stress [26]. Additionally, it has been shown that a pro-oxidant can promote Keap1 S-glutathionylation and subsequent Nrf2 activation [27].

Nrf2, a master transcription factor, coordinates the activation of a variety of cytoprotective genes in response to oxidative stress. The transcriptional activity of Nrf2 is primarily regulated by Keap1 [28]. Under normal conditions, Keap1 sequesters Nrf2 in the cytoplasm and targets it for degradation by the proteasome. However, oxidative and electrophilic stimuli can disrupt the interaction between Nrf2 and Keap1 by modifying specific cysteine residues of Keap1. This disruption allows Nrf2 to escape degradation and translocate into the nucleus, where it activates the expression of numerous antioxidant proteins, including enzymes involved in GSH biosynthesis and maintenance [28,29]. Studies have reported that chemical inducers of Nrf2 can mitigate β-cell damage induced by streptozotocin [30], while Keap1-knockout mice with Nrf2 induction in pancreatic β-cells show significant suppression of oxidative and nitrosative stress-induced dysfunction [31]. These findings suggest that Nrf2 induction plays a protective role against reactive species damage in pancreatic β-cells. Together, the Keap1-Nrf2 system is considered a critical defense pathway for the physiological and pathological protection of pancreatic β-cells. In this study, we investigated the proposition that GSH might augment the S-glutathionylation of Keap1. Consequently, this process leads to the activation of Nrf2 and promotes the expression of genes linked to antioxidative and protective effects in β-cells under an OsG environment.

## 2. Materials and Methods

### 2.1. In Vivo Studies

The development of a prediabetic rat model and the drug administration process were conducted in accordance with previously established guidelines [6]. Sprague-Dawley rats weighing 200 ± 10 g were obtained from the Chinese Academy of Medical Sciences in Shanghai, China. The rats were housed in a controlled environment at a temperature of 22 ± 1 °C, with a 12-h light-dark cycle regulated automatically. They were given unrestricted access to rat chow and water. The Institutional Animal Care and Use Committee of Wenzhou Medical University approved the experimental protocol, and all experiments were conducted following the guidelines for animal experimentation.

The LOsG group and the sham group received different treatments. The LOsG group was administered 6 g/kg of glucose, and the sham group was given distilled water (2 mL/100 g body weight) every 6 h via gavage for a total of 38 days. Additionally, to assess the preventive effect of GSH on LOsG-induced ROS homeostasis disorder, the LOsG.TdGSH group underwent simultaneous subcutaneous injection of GSH at a dose of 50 mg/kg/6 h, following the same challenge as the LOsG group described above.

### 2.2. Cell Culture and Treatments

RIN-m5F cells, a pancreatic β-cell line derived from rats, were procured from the cell bank of the Chinese Academy of Sciences (Shanghai, China). The cells were cultivated in RPMI-1640 medium supplemented with 1% penicillin/streptomycin and 10% fetal bovine serum in a 5% CO_2_ incubator at 37 °C. During a 6-day experimental period, the cells were incubated under different conditions: (1) constant normal glucose (NG) concentration of 5.5 mmol/L and (2) oscillating glucose (OsG), alternating between 24-h periods of 5.5 mmol/L and 25 mmol/L glucose media. The OsG condition was further divided into two subgroups, one with GSH (13 mmol/L) supplementation and one without supplementation. Both glucose (S11022) and GSH (S20186) were procured from Shanghai Yuanye Bio-Technology Co., Ltd. (Shanghai, China). Fresh medium was replenished daily. Samples were collected from the OsG conditions while the cells were exposed to the 25 mmol/L glucose medium. In some experiments, an additional treatment involving 5 μM ML385 (Topscience, Shanghai, China) was administered to the cells.

### 2.3. Pancreatic Islet Isolation and Primary Culture

Islets were procured from Sprague-Dawley rats using a manual handpicking method after pancreata digestion with collagenase P (Sigma-Aldrich, St. Louis, MO, USA) through the bile duct, as previously described [32]. The isolated islets were cultured in RPMI-1640 medium supplemented with 10% fetal bovine serum and 5.5 mmol/L glucose for 24 h. Subsequently, they were treated with the following media for 6 days, similar to the treatment regimen for RIN-m5F cells mentioned above: (1) continuous NG, (2) OsG, and (3) OsG + GSH (13 mmol/L). The culture medium was refreshed daily.

### 2.4. Glucose-Stimulated Insulin Secretion (GSIS) Assay

The islet function was evaluated using the previously reported GSIS method [33]. Following drug treatments, the islets were collected to perform the GSIS assay. In brief, 150 islet equivalents (IEQs) were initially incubated in KRBH buffer (composed of 129 mM NaCl, 4.7 mM KCl, 1.2 mM KH_2_PO_4_, 2.5 mM CaCl_2_, 1.2 mM MgSO_4_, 5 mM NaHCO_3_, 10 mM Hepes, and 0.1% BSA) supplemented with 2.8 mM glucose for 1 h. Subsequently, the islets were placed in a KRBH solution containing 2.8 mM glucose (low) and incubated at 37 °C for 1 h, and the supernatants were collected. Following this, the islets were washed thrice with KRBH and then incubated in KRBH solution spiked with 16.7 mM glucose (high) at 37 °C for 1 h, and the supernatants were collected again. The insulin content in the supernatant samples was measured using the Ultrasensitive Insulin ELISA kit (Mercodia, Uppsala, Sweden) following the manufacturer’s instructions. The stimulation index was calculated using the following equation: Stimulation index = C_high glucose_/C_low glucose._

C_high-glucose_ was the secreted insulin concentration of islets under high-glucose stimulation, and C_low-glucose_ was the secreted insulin concentration of islets under low-glucose stimulation.

### 2.5. RNA Isolation and Real-Time Quantitative PCR

Total RNA was extracted using TRIzol and reverse transcribed into cDNA employing the HiScript^®^ III RT SuperMix (Vazyme, Nanjing, China). Subsequently, mRNA expression was quantified using the ChamQ Universal SYBR qPCR Master Mix (Vazyme) on an ABI StepOne Plus Real-Time PCR system (Applied Biosystems, Foster City, CA, USA). The data were analyzed utilizing the comparative method (2^−ΔΔCT^), with β-actin serving as the internal control, and the results were expressed relative to those in the NG group. The sequences of the primers used in this study are listed in Table 1.

### 2.6. Western Blotting

Protein samples were extracted from whole cells using RIPA buffer (Beyotime Biotechnology, Shanghai, China) supplemented with a cocktail of protease inhibitors (Thermo Fisher Scientific, Waltham, MA, USA). The extraction of cytosolic and nuclear proteins was performed using respective extraction kits following the manufacturer’s instructions (Beyotime). The protein concentration was determined using a BCA Protein Assay Kit (Beyotime). Equal amounts of protein (20 μg) from each sample were separated by SDS-PAGE and were subsequently transferred onto a PVDF membrane (Millipore, Burlington, MA, USA). Antigens were visualized by sequential treatment with specific antibodies, secondary antibodies conjugated to horseradish peroxidase, and an enhanced chemiluminescence substrate kit (Millipore Corporation, Burlington, MA, USA). Primary antibodies against insulin (no. 38355) were obtained from SAB^®^ (Greenbelt, MD, USA). Antibodies against MafA (no. 79737) were obtained from CST (Danvers, MA, USA). Antibodies against Grx1 (no. 15804-1-AP), Keap1 (no. 10503-2-AP), Nrf2 (no. 16396-1-AP), Pdx1 (no. 20989-1-AP), HO-1 (no. 10701-1-AP), NQO-1 (no. 67240-1-lg), and GAPDH (no. 10494-1-AP) were purchased from ProteinTech (Wuhan, China). Antibodies against Ngn3 (bs-0922R) were obtained from Bioss (Beijing, China). Antibodies against GCLC (AF6969) were obtained from Beyotime Biotechnology (Shanghai, China). Antibodies against β-actin (AP0060) were procured from Bioworld (Louis Park, MN, USA).

To assess the total S-glutathionylation of whole-cell proteins, cell lysis buffer was supplemented with 10 mM N-Ethylmaleimide (NEM) to block unreacted thiols. The protein samples were then resolved by SDS-PAGE under non-reducing conditions. Parallel reactions were also conducted under reducing conditions in the presence of 10 mM dithiothreitol (DTT). The intensities of bands were quantified by densitometry using the ImageJ software (version 1.60; NIH, Bethesda, MD, USA).

### 2.7. Co-Immunoprecipitation and Determination of S-Glutathionylation of Keap1

Cells were lysed using NP40 cell lysis buffer (Beyotime). The lysate obtained was then subjected to centrifugation and subsequently incubated with antibodies specific to Keap1 or Flag (no. 14793, CST, Danvers, MA, USA) overnight at 4 °C. Protein G magnetic beads (ThermoFisher) were used to purify the immune complexes at 4 °C for 2 h, followed by centrifugation and washing with NP40 cell lysis buffer for 4–5 times. The immunoprecipitated protein was further analyzed by western blotting using an anti-Nrf2 or anti-GSH antibody (ab19534, Abcam, Cambridge, UK).

For the determination of S-glutathionylated Keap1, cells were lysed in ice-cold lysis buffer supplemented with 10 mM NEM. The supernatants of the whole-cell lysates were incubated with an antibody specific to GSH, subsequently purified by protein G magnetic beads, and then resolved by SDS-PAGE under non-reducing conditions, followed by incubation with a Keap1 antibody.

### 2.8. Construction of Keap1 Cysteine Mutants

The full-length rat Keap1 construct, contained in the pCMV-3×flag plasmid (P44720), was procured from MiaoLingBio (Wuhan, China). It served as the template for conducting site-directed mutagenesis. The DNA sequencing analysis validated the successful single-site mutation of Cys151, Cys273, or Cys288 to alanine. The expression plasmids were transfected into cells using the Lipofectamine 3000 reagent (Invitrogen, Carlsbad, CA, USA).

### 2.9. Immunofluorescence Staining

Paraffin-embedded sections of the pancreas of rats were deparaffinized and rehydrated, followed by antigen retrieval in 10 mmol/L sodium citrate buffer. After blocking in 5% goat serum (Beyotime) for 30 min, the sections were incubated with antibodies against insulin (no. 66198-1-Ig, ProteinTech), Nrf2 (no. 16396-1-AP, ProteinTech), HO-1 (no. 10701-1-AP, ProteinTech), and Grx1 (no. A5315, ABclonal, Wuhan, China) at 4 °C overnight, followed by incubation with Alexa Fluor 488- or 594-conjugated secondary antibodies (1:400 dilution, Molecular Probes, Waltham, MA, USA). DAPI was used for nuclei staining.

For immunostaining of cultured RIN-m5F cells, the cells were fixed in 4% formaldehyde for 15 min and then incubated in 0.5% Triton-X100 (MACKLIN, Shanghai, China) for 15 min. After blocking with 3% bovine serum albumin at room temperature for 1 h, the cells were incubated overnight at 4 °C with an Nrf2 antibody. This was followed by a 1-h incubation with an Alexa Fluor 488-conjugated secondary antibody (1:500 dilution, Molecular Probes) after washing. Negative control sections were treated with PBS instead of a primary antibody. The antibodies were visualized using a Leica DM6000B fluorescence microscope (Wetzlar, Germany) with the Leica Application Suite X software (3.6.1.23246).

### 2.10. GSH/GSSG Assay

The GSH/GSSG (oxidized GSH) ratio was determined using a GSH/GSSG assay kit according to the manufacturer’s instructions (Beyotime). Briefly, the cells were subjected to two rapid freeze-thaw cycles using liquid nitrogen and a water bath at 37 °C. The resulting supernatants were collected, and the total GSH [GSH + GSSG] level was determined by measuring the production of 2-nitro-5-thiobenzoic acid at 412 nm in the presence of 5,5′-dithio-bis-2-nitrobenzoic acid, NADPH, and GSH reductase. GSSG was quantified by removing GSH from the samples using the reagents provided in the kit and analyzing the derivatized samples as described above for total GSH. Standard curves were generated for both GSH and GSSG, and the concentration of GSH was calculated by subtracting the GSSG concentration from the total GSH concentration. Finally, the GSH/GSSG ratio was obtained by plotting GSH against GSSG.

### 2.11. Detection of Mitochondrial ROS Level

Cells were washed with PBS and incubated with DCFH-DA (Beyotime) at a final concentration of 10 μM at 37 °C in the dark for 30 min. The fluorescence intensity of samples was determined using a FACSCanto II flow cytometer (BD, Mountain View, CA, USA).

### 2.12. Apoptosis Assay

Apoptosis was assessed using the APC Annexin V Staining Kit (Tonbo Biosciences, San Diego, CA, USA). Following the designated treatments stated above, the cells were collected through centrifugation. The cell pellets were then rinsed with staining buffer and resuspended in binding buffer. Subsequently, the cells were incubated in the dark for 15 min with APC Annexin V and 7-AAD. Finally, the samples were washed to remove extra stains and analyzed by FACSCanto II flow cytometry to determine the percentage of apoptosis.

### 2.13. Statistical Analysis

The data from at least three independent experiments were analyzed and presented as the mean ± standard error of the mean (SEM). The sample size was calculated by using the Sample Size Calculator (https://www.surveysystem.com/sscalc.htm (accessed on 25 February 2022)) and conformed to the pre-specified effect size. Group comparisons were analyzed using ordinary one-way ANOVA followed by Tukey’s multiple comparisons test when appropriate. Statistical analysis was conducted using the GraphPad Prism 8.0.1 software (GraphPad Software, Boston, MA, USA). A value of *p* < 0.05 was considered statistically significant.

## 3. Results

### 3.1. GSH Prevents the Inhibition of Nrf2, Grx1, and HO-1 Expressions in Islets of Rats Induced by LOsG

**Figure 1:**
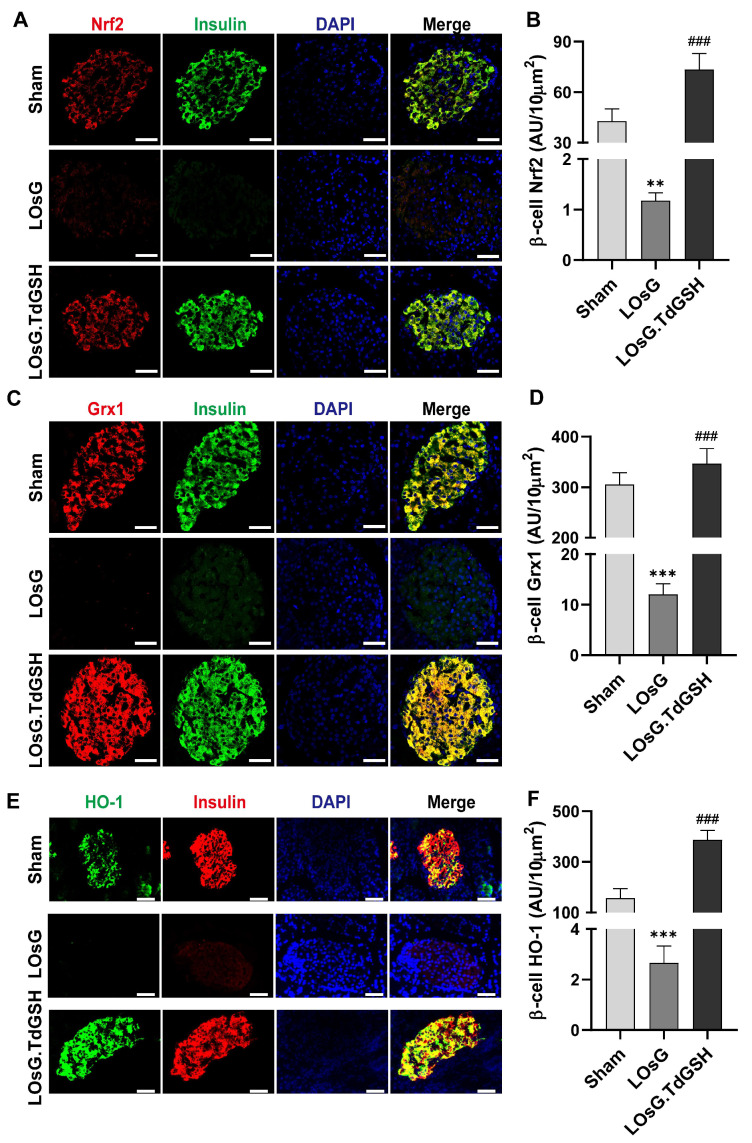
GSH prevents LOsG-induced inhibition of Nrf2, Grx1, and HO-1 expressions in islets of rats (*n* = 5–6 rats/group). (**A**) Representative images of immunofluorescence staining for Nrf2 (red) and insulin (green). The nuclei were stained with DAPI (blue). Scale bars, 100 μm. (**B**) Quantification of Nrf2 staining (presented as arbitrary unit/mm^2^). (**C**) Representative images showing immunostaining for Grx1 (red), insulin (green), and DAPI (blue). Scale bars, 100 μm. (**D**) Quantification of Grx1 staining. (**E**,**F**) Representative immunostaining for HO-1 (green), insulin (red), and DAPI (blue) and quantification of HO-1. Scale bars, 50 μm. Data shown are mean ± SEM. ** *p* < 0.01, *** *p* < 0.001 vs. sham group; ^###^ *p* < 0.001 vs. LOsG group.

Our previous in vivo studies have demonstrated that LOsG treatment leads to oxidative stress and dedifferentiation of β-cells, resulting in insulin-secreting functional failure. However, the deleterious effects of LOsG/ROS-induced β-cells can be mitigated by the administration of exogenous GSH [6]. To investigate the underlying mechanisms of LOsG and GSH effects through in vivo pancreatic islets, we determined the roles of Nrf2, which regulates an array of antioxidant genes in the face of oxidative stress [29], suppresses oxidative damage of pancreatic islets, and strongly restores insulin secretion in diabetic conditions [31]. Interestingly, LOsG significantly reduced the expressions of insulin and Nrf2 in β-cells. The timely administration of GSH (TdGSH) completely prevented the decreases in both insulin and Nrf2 induced by LOsG in β-cells (Figure 1A,B). Consequently, the expressions of Nrf2-related downstream signaling genes responsible for antioxidant defense factors, including glutaredoxin 1 (Grx1) and heme oxygenase-1 (HO-1), were significantly decreased in β-cells of rats treated with LOsG. Nevertheless, this effect was reversed upon treatment with GSH (Figure 1C–F). These findings indicate that exogenous GSH can effectively prevent β-cell damage caused by LOsG, likely through the regulation of redox balance via the Nrf2 pathway.

### 3.2. GSH Prevents OsG-Induced β-Cell Failure

**Figure 2:**
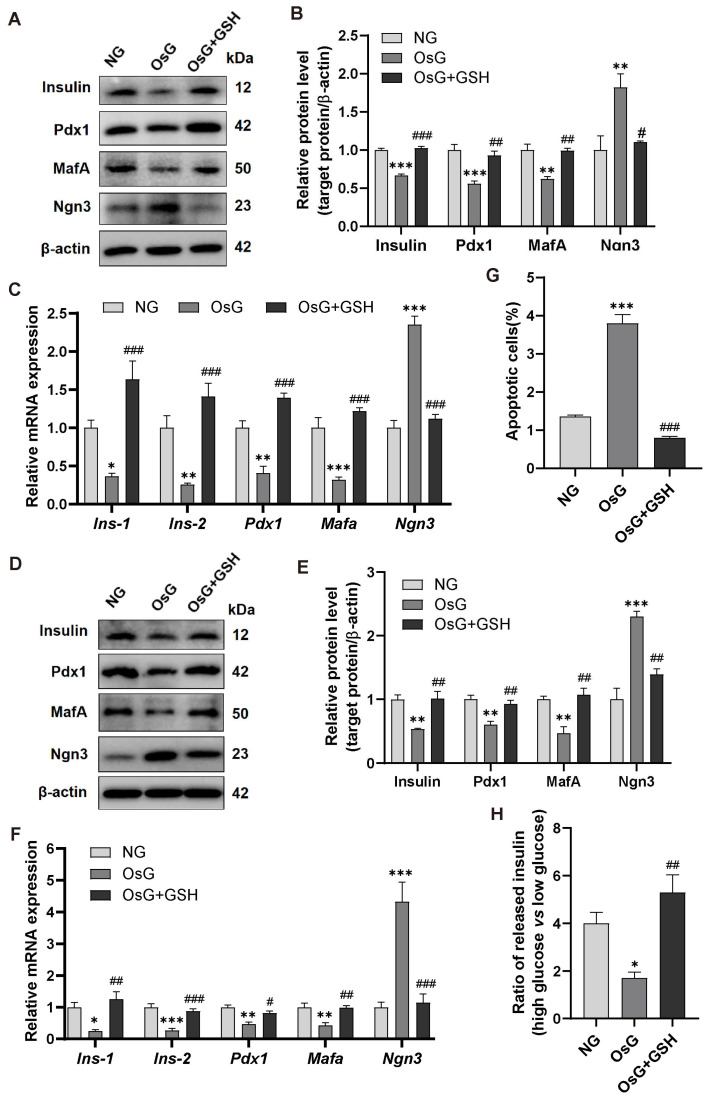
GSH prevents OsG-induced β-cell failure. Representative western blots and densitometry analysis (**A**,**B**) as well as mRNA levels of (**C**) insulin, Pdx1, MafA, and Ngn3 in RINm5F cells (*n* = 3–5). Western blotting analysis and quantification (**D**,**E**) along with mRNA levels of (**F**) insulin and its related gene expressions in primary islets of rats (*n* = 3–5). (**G**) The percent of apoptotic cells in RINm5F cells (*n* = 4). (**H**) Glucose-stimulated insulin secretion of primary islets of rats (*n* = 4). Data shown are mean ± SEM. (**B**–**H**): * *p* < 0.05, ** *p* < 0.01, *** *p* < 0.001 vs. NG group; ^#^ *p* < 0.05, ^##^ *p* < 0.01, ^###^ *p* < 0.001 vs. OsG group.

To further examine the impact of OsG and the protective effect of GSH on β-cell, we performed in vitro experiments utilizing RINm5F cells and isolated primary rat islets. Consistent with the results from the in vivo investigation [6], exposure to OsG for 6 days in these cells led to a significant decrease in the expressions of insulin and its transcription factors Pdx1 and MafA. Additionally, the progenitor cell marker Ngn3, indicating dedifferentiation of β-cell, showed an increase in expression. Notably, these changes were reversed upon treatment with GSH (Figure 2A–F). Furthermore, it was observed that GSH inhibited the apoptosis of RINm5F cells induced by OsG (Figure 2G). As a result, GSH demonstrated its capability to restore β-cell functionality and mitigate the adverse impact of OsG on glucose-stimulated insulin secretion in primary rat islets, as evidenced by the ratio of released insulin under high/low glucose stimulation (Figure 2H). Collectively, these findings suggest that the administration of exogenous GSH can successfully prevent β-cell failure triggered by OsG, which is consistent with our previous in vivo studies [6].

### 3.3. GSH Activates Nrf2 Pathway in OsG-Treated β-Cells

**Figure 3:**
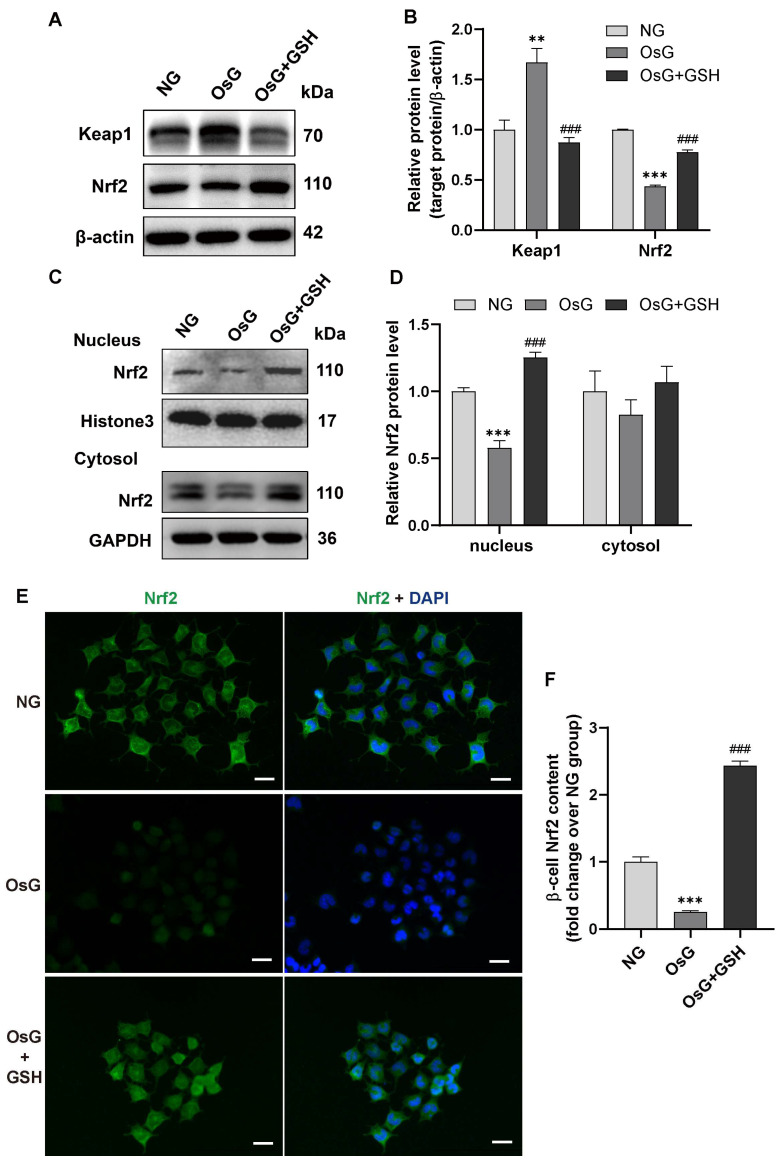
GSH activates the Nrf2 pathway in OsG-treated RINm5F cells. (**A**) Representative western blots depicting Keap1 and Nrf2 protein levels and (**B**) densitometry analysis of the same proteins (*n* = 3–4). (**C**,**D**) Western blotting analysis of Nrf2 protein levels in nuclear and cytosolic extracts prepared from RINm5F cells. Histone H3 and GAPDH were used as normalization controls for nuclear and cytosolic proteins, respectively (*n* = 3). (**E**) Immunofluorescence staining of RINm5F cells using an antibody against Nrf2 (green) and DAPI (blue). Scale bars, 100 μm. (**F**) Quantification of Nrf2 staining (*n* = 6). Data shown are mean ± SEM. (**B**,**D**,**F**): ** *p* < 0.01, *** *p* < 0.001 vs. NG group; ^###^ *p* < 0.001 vs. OsG group.

To investigate the possible participation of Nrf2, a transcription factor that remains inactive by forming a complex with Keap1 in the cytoplasm but disassociates from Keap1 and relocates to the nucleus during oxidative stress [28,29], in the cytoprotective effects of GSH on β-cell function, we analyzed the expression and localization of Nrf2 in RINm5F cells treated with OsG, both with and without GSH. As presented in Figure 3A,B, the expression of Keap1 was noticeably elevated, while the level of Nrf2 was significantly reduced in cells treated with OsG compared to those treated with NG. However, this effect was reversed upon GSH treatment. Western blotting analysis of cytoplasmic and nuclear protein extracts indicated that treatment with GSH enhanced the nuclear accumulation of the Nrf2 protein compared to cells treated with OsG alone (Figure 3C,D). Furthermore, the immunofluorescence assay revealed intensified nuclear staining of Nrf2 in cells after being treated with GSH (Figure 3E,F), indicating that Nrf2 was activated in response to GSH supplementation.

### 3.4. GSH Promotes Nrf2-Related Antioxidant Enzyme Expression to Attenuate Oxidative Stress in OsG-Treated β-Cells and Primary Islets

To investigate the influence of Nrf2 on the regulation of antioxidant genes carrying antioxidant response elements upon redox stress, we subsequently examined the expression of Nrf2-targeted antioxidant defense enzymes, including the glutamate-cysteine ligase catalytic subunit (GCLC), Grx1, heme oxygenase-1 (HO-1), and NADPH quinone oxidoreductase 1 (NQO1) [29].

**Figure 4:**
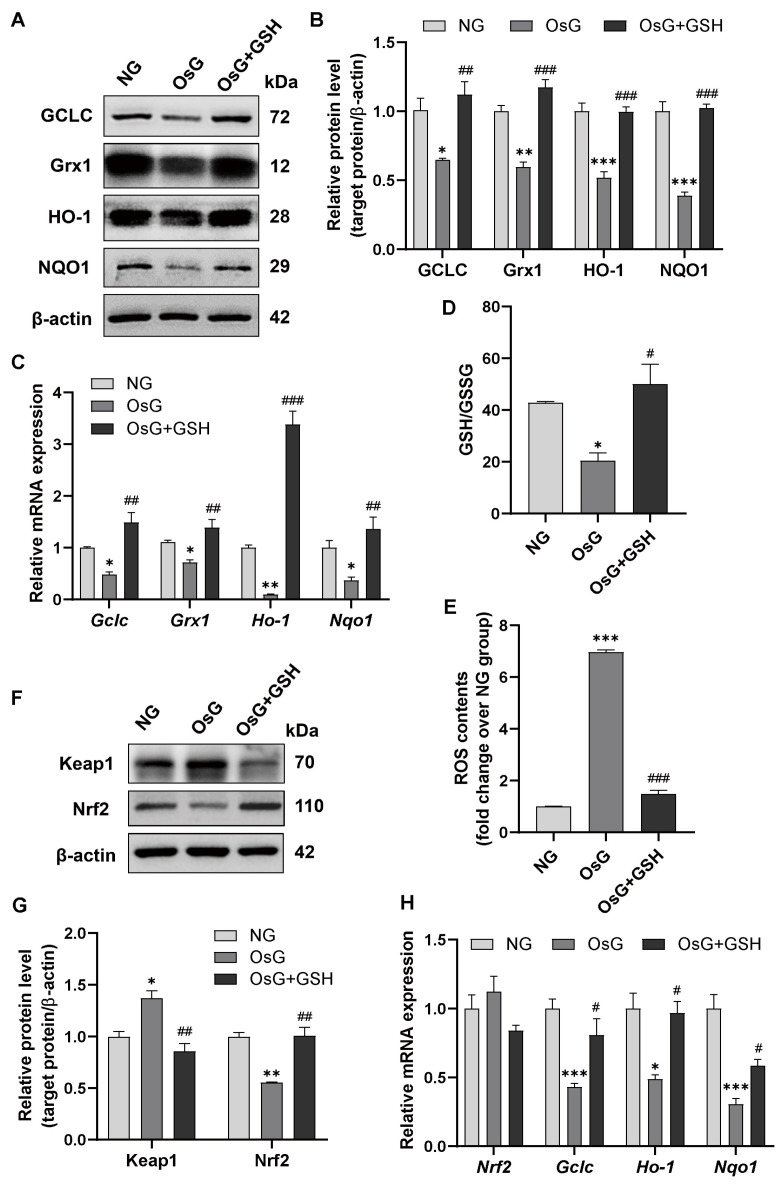
GSH-mediated promotion of Nrf2-related antioxidant enzyme expression mitigates oxidative stress in β-cells and primary islets treated with OsG. (**A**,**B**) Representative western blots and densitometry analysis depicting the protein levels of GCLC, Grx1, HO-1, and NQO1 in RINm5F cells (*n* = 3–4). (**C**) mRNA levels of GCLC, Grx1, HO-1, and NQO1 in RINm5F cells (*n* = 3–4). (**D**) GSH/GSSG level in RINm5F cells (*n* = 3). (**E**) ROS contents in RINm5F cells (*n* = 3). (**F**,**G**) Western blotting analysis and quantification of Keap1 and Nrf2 in primary islets of rats (*n* = 3). (**H**) The mRNA levels of Nrf2 and its target genes in primary islets of rats (*n* = 3–5). Data shown are mean ± SEM. (**B**–**H**): * *p* < 0.05, ** *p* < 0.01, *** *p* < 0.001 vs. NG group; ^#^ *p* < 0.05, ^##^ *p* < 0.01, ^###^ *p* < 0.001 vs. OsG group.

The mRNA and protein levels of GCLC, Grx1, HO-1, and NQO1 in RINm5F cells exposed to OsG were significantly reduced. As expected, this effect was reversed when the cells were treated with GSH (Figure 4A–C). Furthermore, the administration of GSH noticeably elevated the GSH/GSSG ratio, thereby mitigating the increase in ROS levels caused by OsG (Figure 4D,E). A similar phenomenon was observed in primary rat islets treated with OsG. Treatment with GSH led to decreased expression of Keap1 and an increased level of Nrf2 protein (Figure 4F,G). Consequently, GSH counteracted the decrease in mRNA levels of GCLC, HO-1, and NQO1 induced by OsG (Figure 4H). These findings suggest that Nrf2-related antioxidant enzymes play a crucial role in mediating the inhibitory effects of GSH on OsG-induced oxidative stress in β-cells.

### 3.5. Inhibition of Nrf2 Abrogates the Cytoprotective Effects of GSH in OsG-Treated β-Cells

**Figure 5:**
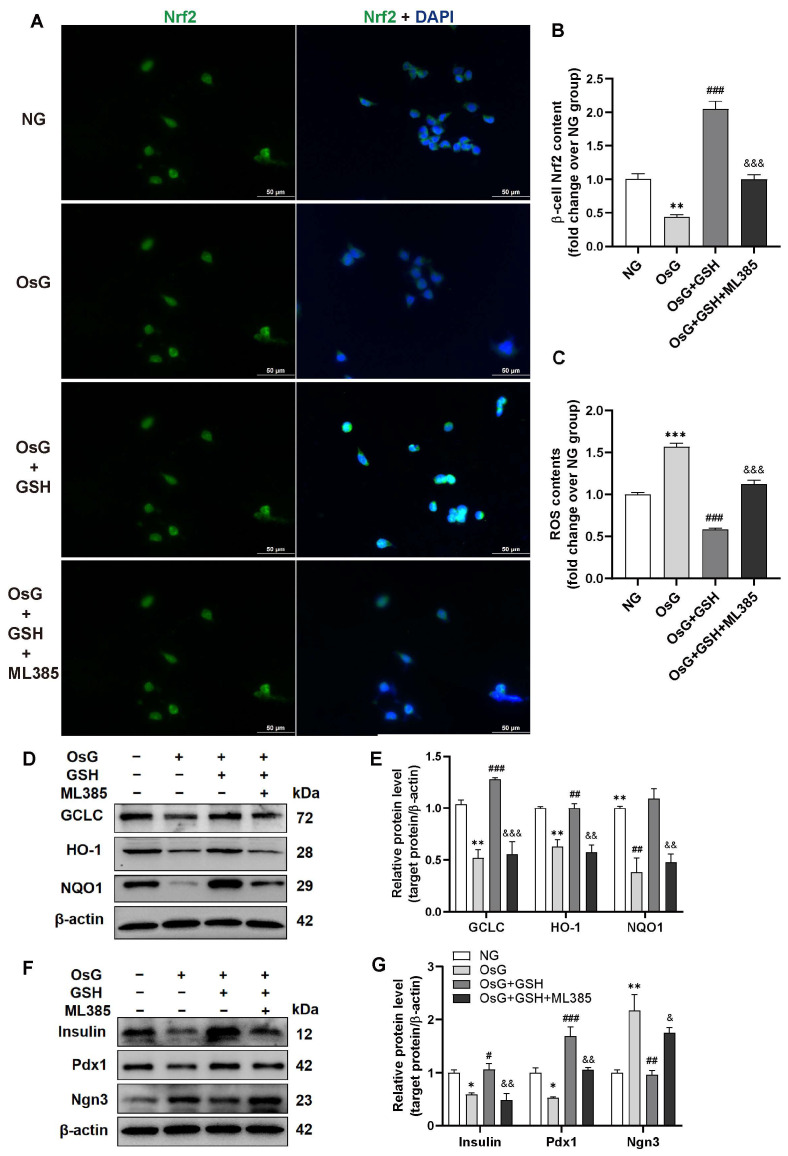
Blocking Nrf2 activity eliminates the protective effects of GSH in β-cells treated with OsG. RINm5F cells were cultured under oscillating glucose conditions with or without GSH for 4 days, followed by treatment with or without ML385 for an additional 2 days. (**A**) Immunofluorescence staining of Nrf2 (green) and DAPI (blue) was performed on RINm5F cells. Scale bars, 50 μm. (**B**) Quantification of Nrf2 staining (*n* = 4). (**C**) Measurement of ROS levels in RINm5F cells (*n* = 3). Representative western blots (**D**) and densitometry analysis of (**E**) GCLC, HO-1, and NQO1 protein levels (*n* = 3). Representative western blots (**F**) and densitometry analysis of (**G**) insulin, Pdx1, and Ngn3 protein levels (*n* = 3). Data shown are mean ± SEM. * *p* < 0.05, ** *p* < 0.01, *** *p* < 0.001 vs. NG group; ^#^ *p* < 0.05, ^##^ *p* < 0.01, ^###^ *p* < 0.001 vs. OsG group; ^&^ *p* < 0.05, ^&&^ *p* < 0.01, ^&&&^ *p* < 0.001 vs. OsG + GSH group.

In order to determine whether the downregulation of oxidative stress induced by GSH depends on the activation of the Nrf2 pathway, we treated RIN-m5F cells with the Nrf2 inhibitor ML385, which inhibits the activity of Nrf2 by binding to Neh1, a CNC-bZIP domain that allows Nrf2 to heterodimerize with small Maf proteins, blocking Nrf2 transcriptional activity [34,35]. Our observations revealed that inhibiting Nrf2 activity abolished the GSH-mediated nuclear translocation of Nrf2 (Figure 5A,B) and suppression of ROS production (Figure 5C) induced by OsG in β-cells. Furthermore, the expression of Nrf2-targeted genes GCLC, HO-1, and NQO1, as well as insulin and Pdx1, were significantly reduced, while the expression of Ngn3 was remarkably elevated in OsG + GSH-treated cells in the presence of ML385 compared to OsG + GSH-incubated cells without ML385 (Figure 5D–G). Together, these results indicate that the cytoprotective effects of GSH in OsG-treated β-cells are partially mediated by the Nrf2 pathway.

### 3.6. GSH Increases the S-Glutathionylation of Keap1 to Activate the Nrf2 Pathway in OsG-Treated β-Cells

**Figure 6:**
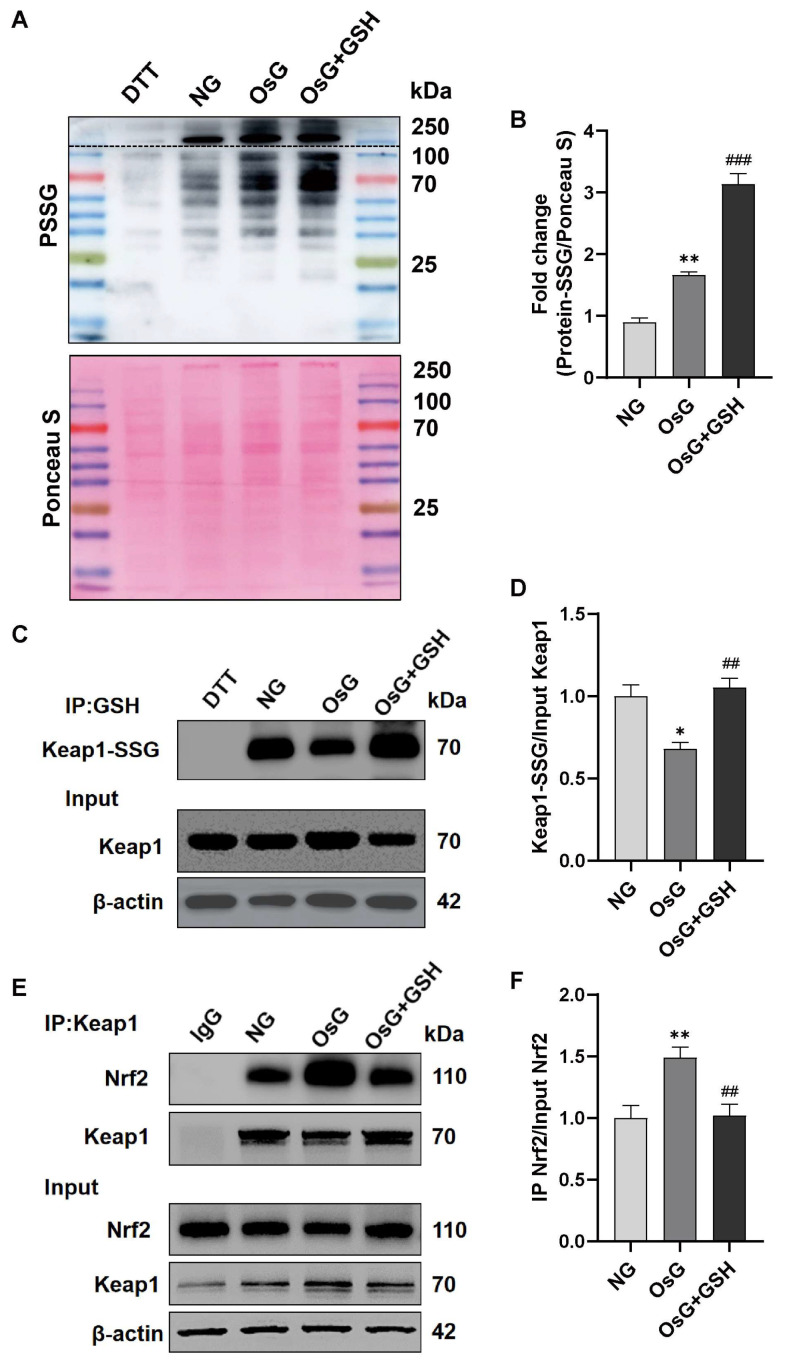
GSH enhances the S-glutathionylation of Keap1, thereby activating the Nrf2 pathway in RINm5F cells treated with OsG. (**A**) Total S-glutathionylated protein level (PSSG) in cell lysates, with DTT serving as the negative control. The top blot above the dashed line displays the PSSG bands under short exposure, and the bottom blot shows the PSSG bands after long exposure. The next figure shows Ponceau S staining of the membrane prior to incubation with an anti-glutathione antibody. (**B**) Semi-quantitative analysis of PSSG blots normalized by Ponceau S staining is presented in the graph (*n* = 3). (**C**) Cell lysates were immunoprecipitated with an anti-glutathione antibody and blotted with an anti-Keap1 antibody. An aliquot of the total lysate was analyzed for Keap1 and β-actin expression. (**D**) Quantification of S-glutathionylated Keap1 normalized by total Keap1 protein (*n* = 3). (**E**) Cell lysates were subjected to immunoprecipitation using an anti-Keap1 antibody or an anti-IgG antibody (negative control), followed by probing with anti-Nrf2 and anti-Keap1 antibodies. An aliquot of the total lysate was analyzed for Nrf2, Keap1, and β-actin expression. (**F**) Quantitative analysis of Nrf2 immunoprecipitated by Keap1 and normalized by total Nrf2 protein (*n* = 3). Data shown are mean ± SEM. (**B**,**D**,**F**): * *p* < 0.05, ** *p* < 0.01 vs. NG group; ^##^ *p* < 0.01, ^###^ *p* < 0.001 vs. OsG group.

Next, we investigated the potential involvement of Keap1 S-glutathionylation, a process associated with Nrf2 activation [26], in the β-cell injury induced by OsG. Initially, we quantified the level of total S-glutathionylated protein (PSSG) in β-cells. As anticipated, exposure to OsG markedly elevated the PSSG level. Interestingly, the administration of GSH further augmented the overall levels of S-glutathionylated proteins (Figure 6A,B). Subsequently, we isolated the S-glutathionylated proteins using an anti-GSH antibody and validated the presence of S-glutathionylated Keap1 (Keap1-SSG) by using western blotting. Surprisingly, despite the increase in total PSSG, we observed a decrease in Keap1-SSG in cells induced by OsG compared to cells in an NG environment. However, treatment with GSH significantly increased the S-glutathionylation of Keap1 in the OsG-incubated cells (Figure 6C,D). In addition, co-immunoprecipitation analysis demonstrated that the treatment with OsG promoted the association between Keap1 and Nrf2. Nevertheless, this interaction was attenuated following the addition of GSH (Figure 6E,F). These results indicate that GSH application provides protection against OsG-induced damage in β-cells, predominantly by reinstating the redox equilibrium through the S-glutathionylation of Keap1 to activate the Nrf2 pathway.

### 3.7. The S-Glutathionylation of Keap1 at Cys273 and Cys288 Caused the Activation of the Keap1/Nrf2 Pathway in GSH-Treated β-Cells under OsG Condition

**Figure 7:**
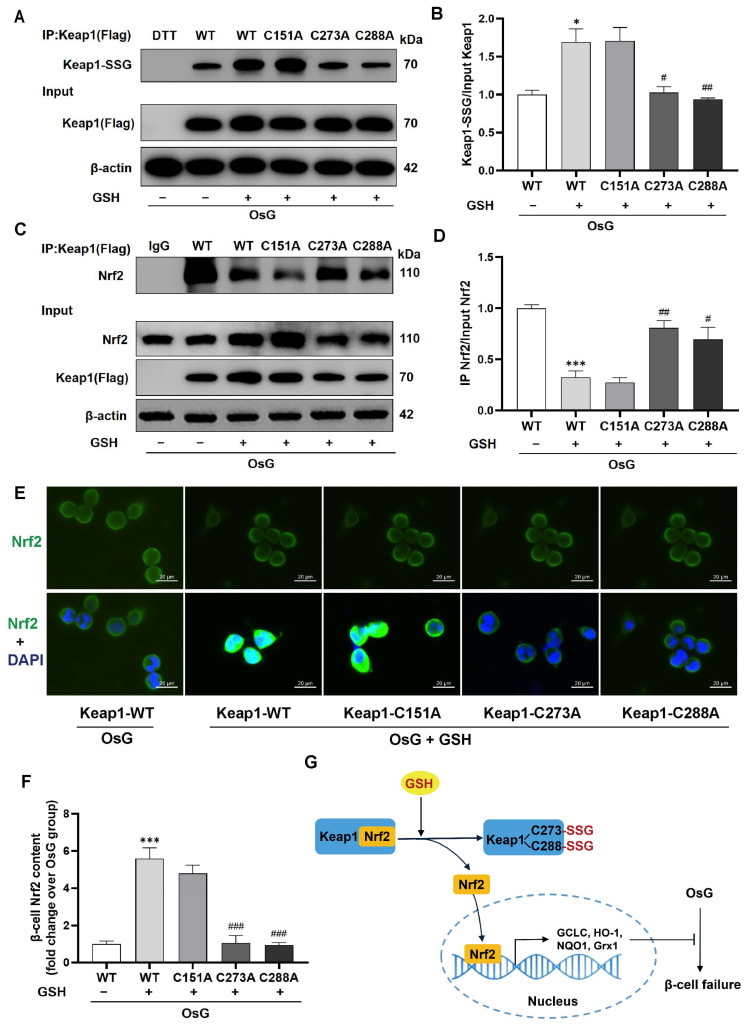
Inhibition of Keap1 S-glutathionylation by Cys273 and Cys288 mutations suppressed the activation of the Nrf2 pathway in GSH-treated β-cells under OsG conditions. RINm5F cells were cultured with oscillating glucose with or without GSH for 72 h prior to transfection, followed by plasmid transfection of Keap1-WT or mutated Keap1 at Cys151, Cys273, and Cys288 for 24 h and by OsG treatment in the presence or absence of GSH for an additional 72 h. (**A**) Cell lysates were immunoprecipitated using an anti-Flag antibody, and the immunoprecipitated proteins were analyzed by immunoblotting with anti-glutathione antibody (DTT served as a negative control). A portion of the total lysate was also analyzed using an anti-Flag antibody. (**B**) The level of S-glutathionylated Keap1 was quantified and normalized to the total Keap1 protein (*n* = 3). (**C**) Cell lysates were immunoprecipitated using an anti-Flag antibody and then immunoblotted with an anti-Nrf2 antibody. The total lysates were analyzed for the expression of Nrf2, Keap1 (Flag), and β-actin. (**D**) Quantitative analysis of Nrf2 immunoprecipitated by Keap1 and normalized to the total Nrf2 protein (*n* = 3). (**E**) Immunofluorescence staining was performed on cells using antibodies against Nrf2 (green) and DAPI (blue). Scale bars, 20 μm. (**F**) Quantification of Nrf2 staining (*n* = 10). Data shown are mean ± SEM. (**B**,**D**,**F**): * *p* < 0.05, *** *p* < 0.001 vs. Keap1-WT + OsG group; ^#^ *p* < 0.05, ^##^ *p* < 0.01, ^###^ *p* < 0.001 vs. Keap1-WT + OsG + GSH group. (**G**) The schematic diagram illustrates that GSH S-glutathionylates Keap1 at Cys273 and Cys288, leading to the dissociation of Nrf2 from Keap1. This, in turn, facilitates the translocation of Nrf2 into the nucleus, where it activates the transcription of antioxidant genes, thereby inhibiting OsG-induced β-cell failure.

The most straightforward approach to validate the functional significance of post-translational modification is to employ mutagenesis techniques for substituting an amino acid residue that undergoes posttranslational modification with one incapable of undergoing the relevant chemical transformation. To identify the S-glutathionylated cysteine residue of Keap1, we transfected Keap1-encoding plasmids in which Cys151, Cys273, or Cys288 was mutated to alanine (C151A, C273A, or C288A), as well as wild-type (WT) plasmids, into β-cells. In conditions of OsG, GSH still caused an increase in the S-glutathionylation of Keap1 in cells transfected with WT and C151A plasmids (Figure 7A,B). However, this increase was not observed in cells transfected with C273A or C288A plasmids. Furthermore, after the mutation of Keap1 at Cys273 or Cys288, GSH failed to induce the dissociation of Nrf2 from Keap1 (Figure 7C,D) and subsequent nuclear translocation (Figure 7E,F) under OsG treatment. These findings suggest that the activation of Nrf2 by GSH depends on the S-glutathionylation of the Keap1 Cys273 and Cys288 residues in β-cells treated with OsG.

## 4. Discussion

Hyperglycemia-induced increases in ROS contribute to the activation of detrimental pathways, leading to the initiation and progression of diabetic complications [36]. The pancreatic β-cell, compared to other tissues, possesses a relatively weaker antioxidant defense system, making it more susceptible to ROS-induced damage [13,14,15]. Our previous studies have demonstrated that frequent fluctuations in glycemic levels can cause β-cells to develop metabolic memory under exposure to ROS stress. Consequently, this process leads to β-cell dedifferentiation, functional failure, and the development of prediabetes accompanied by hypoinsulinemia and glucose intolerance. The timely administration of the antioxidant GSH effectively prevents these damaging effects, thereby safeguarding against β-cell failure, pancreatic fibrosis, and prediabetes [5,6]. However, the specific mechanisms by which GSH protects β-cells remain unknown. In this study, we established an oscillating glucose model both in vivo and in vitro and found that fluctuating glucose levels lead to the downregulation of downstream genes targeted by Nrf2, resulting in oxidative stress and dysfunction of β-cells. In contrast, the administration of GSH protects against β-cell failure induced by oscillating glucose by promoting the S-glutathionylation of Keap1, which activates Nrf2 and the expression of its targeted genes, including GCLC involved in GSH synthesis, antioxidant enzymes such as Grx1, HO-1, and NQO1.

Several studies have shown that individuals with impaired glucose tolerance or T2D have a decreased ratio of GSH to GSSG in erythrocytes and plasma, as well as elevated levels of oxidative stress [7,8,37]. Interestingly, the intravenous administration of GSH in non-insulin-dependent diabetic patients significantly increased the intraerythrocytic GSH/GSSG ratio and overall glucose uptake [38]. Consistent with these findings, our observations indicated that β-cells treated with OsG exhibited higher levels of ROS accumulation and a decreased GSH/GSSG ratio, which can be attributed to increased GSH consumption, impaired GSH synthesis, and potentially compromised expression of GCLC. On the other hand, the supplementation of GSH facilitated the S-glutathionylation of Keap1 and translocation of Nrf2 into the nucleus, thereby enhancing the expressions of GCLC, Grx1, HO-1, and NQO1, while reducing ROS production. This intervention also prevented β-cell dedifferentiation and apoptosis and improved overall β-cell function under OsG conditions. Additionally, when Nrf2 activity was suppressed, the cytoprotective effects of GSH were abolished, suggesting that Nrf2 partially mediates the beneficial functions of GSH in OsG-treated β-cells.

GSH participates in the process of the S-glutathionylation of proteins by binding itself to cysteine residues. This process plays a critical role in regulating redox signaling, storing GSH, and protecting thiol groups of proteins from irreversible oxidation [22,23,24]. Although S-glutathionylation is commonly associated with oxidative or nitrosative stress, it can also occur under normal physiological conditions. S-glutathionylation can modulate the activity and function of various redox-sensitive proteins, including glyceraldehyde-3 phosphate dehydrogenase (GADPH), protein kinase B (Akt), and protein tyrosine phosphatase 1B (PTP1B) [39,40,41]. Consistent with these findings, our results showed that β-cells exposed to OsG increased levels of PSSG and ROS. Despite the observed increase in PSSG content after the OsG challenge, our data showed that OsG-treated β-cells exhibited significantly decreased Keap1 S-glutathionylation. This attenuation inhibited the activation of the Nrf2 pathway. Therefore, the higher PSSG level induced by OsG was associated with the S-glutathionylation of other proteins but not Keap1. These effects of OsG in β-cells differ from other oxidative stress agents in brain cells. In brain cells with MPTP-induced oxidative stress, cysteine residues of Keap1 undergo S-glutathionylation modification, which leads to a significant increase in Nrf2 activity [26]. The differences observed in these studies could be partly attributed to the heightened sensitivity of pancreatic β-cells to ROS as they rely on ROS as crucial cellular signals [9,10]. This ROS sensitivity potentially distinguishes β-cell from other cell types.

Diabetic patients often exhibit reduced cellular GSH levels [7,8,42,43]. Apart from its role in providing antioxidant protection against oxidative stress caused by hyperglycemia in diabetes, the impact of GSH on the protection of β-cells is still unclear. Here, we have intriguing findings that GSH treatment led to a more significant elevation in PSSG level compared to that of OsG treatment alone, which may be attributed to the administration of GSH increasing the overall GSH and GSSG contents. As a result, this increase was observed to be associated with the promotion of S-glutathionylated Keap1 formation, the translocation of Nrf2 into the nucleus, and the upregulation of Nrf2-targeted antioxidant genes. In line with our findings, a prior investigation exhibited that administering the antioxidant lipoic acid to aged rats led to the stimulation of Nrf2 and consequent accumulation in the nucleus of liver cells. This effect contributed to the augmentation of GSH synthesis through the upregulation of GCLC and GCLM expression [44]. In the present study, administering GSH via subcutaneous injection effectively counteracted the negative impact of LOsG on the expression of Nrf2, Grx1, HO-1, and insulin in islet β-cells. The findings from our in vivo and in vitro studies are in line with previous research, indicating that the Keap1-Nrf2 system plays a crucial role in preventing the onset of diabetes [30,45,46]. Specifically, the induction of Nrf2 through Keap1-conditional knockout in pancreatic β-cells was found to suppress oxidative damage in pancreatic islets and significantly restore insulin secretion in the context of diabetes [31].

Keap1 proteins contain multiple cysteine residues, and among them, Cys151, Cys273, and Cys288 are crucial for the Keap1-dependent ubiquitination of Nrf2 [47,48]. Previous studies have suggested that the S-sulfhydration of Keap1 at Cys151 can promote the dissociation of Nrf2, facilitating its translocation to the nucleus [49,50,51]. In this study, we introduced alanine mutations in Cys151, Cys273, and Cys288 of Keap1, and observed that GSH enhanced Keap1 S-glutathionylation specifically in Cys151-mutant-transfected β-cells. However, it had no effect on cells transfected with Cys273 or Cys288 mutants, indicating the critical role of Cys273 and Cys288 in Keap1 S-glutathionylation. Consequently, GSH failed to promote the dissociation of Nrf2 from Keap1 and its translocation to the nucleus in Cys273 or Cys288 mutant-transfected β-cells. Overall, our findings highlight the significant contribution of Keap1 Cys273 and Cys288 S-glutathionylation in the protective effects of GSH against OsG-induced injury in β-cells.

GSH plays a crucial role in maintaining cellular redox homeostasis and regulating various cell functions such as cell cycle, apoptosis, immunological defense, and pathological abnormality [19,20,52,53]. GSH also interacts with other enzymes and proteins, including glutathione peroxidases and glyoxylases, to engage and regulate cellular processes [20,52]. In this study, our investigations focused on the impact of GSH on the Keap1 S-glutathionylation-related Nrf2 pathway in β-cells challenged by OsG. However, further research is necessary to explore the effects of GSH in other aspects. Moreover, GSH universally participates in the S-glutathionylation modification of cysteine residues in proteins. In the ongoing investigation, a crucial task remains to ascertain the effects of OsG-induced oxidative stress and GSH on protein cysteine residues, along with their implications for proteins beyond Keap1. Furthermore, it is important to explore the potential role of other protein modifications and their contribution, either supportive or detrimental, to the pathological progression of β-cell failure.

## 5. Conclusions

In conclusion, our research provides initial evidence that GSH reverses the specific inhibitions of Keap1 S-glutathionylation at Cys273 and Cys288 caused by OsG, resulting in the reactivation of Nrf2 activity. This reactivation consequently leads to upregulating the expression of targeted antioxidant genes, such as GCLC, HO-1, NQO1, and Grx1, effectively preventing β-cell dedifferentiation and functional failure (Figure 7G). Our findings suggest that the S-glutathionylation of Keap1 may be a key target for the prevention and treatment of β-cell dysfunction in individuals with prediabetes or diabetes. Additionally, the administration of exogenous GSH shows promising potential as an effective strategy to protect against the onset or progression of diabetes.

## Figures and Tables

**Table 1:** Real-time PCR primers.

Target Genes	Forward Primer (5′ to 3′)	Reverse Primer (5′ to 3′)	Accession No.
Ho-1	CCCAGAGGCTGTGAACTCTG	GGGGAAAGCAGTCATGGTCA	NM_012580.2
Nqo1	GAAAGGATGGGAGGTGGTCG	GCTCCCCTGTGATGTCGTTT	NM_017000.3
Gclc	TGATTGAAGGGACACCTGGC	TGTGCTCTGGCAGTGTGAAT	NM_012815.2
Grx1	CGTGGTCTCCTGGAATTTGTG	AAGACCCGAGGAACTGTTCTTG	NM_022278.1
Nrf2	TAGATCTTGGGGTAAGTCGAGA	CTCTTGTCTCTCCTTTTCGAGT	NM_001399173.1
Ins2	TGGAAGCTCTCTACCTGGTGT	GTGCCAAGGTCTGAAGGTCAC	NM_019130.2
Ins1	CTACACACCCAAGTCCCGTC	CCAAGGTCTGAAGATCCCCG	NM_019129.3
Ngn3	GTCAGAGACTGTCACACCCC	TGGAACTGAGCACTTCGTGG	NM_021700.1
MafA	TTCTGGAGAGCGAGAAGTGC	CGCGCTCACAGAAAGAAGTC	XM_017603453.1
Pdx1	CGGACATCTCCCCATACG	AAAGGGAGATGAACGCGG	NM_022852.3
β-actin	TTTAATGTCACGCACGATTTCCC	CCCATCTATGAGGGTTACGC	NM_031144.3

## Data Availability

The raw data supporting the conclusions of this article will be made available by the authors upon request.
