# Correction: Chen et al. Glutathione Induces Keap1 S-Glutathionylation and Mitigates Oscillating Glucose-Induced β-Cell Dysfunction by Activating Nrf2. *Antioxidants* 2024, *13*, 400

**DOI:** 10.3390/antiox15020228

**Published:** 2026-02-10

**Authors:** Xiufang Chen, Qian Zhou, Huamin Chen, Juan Bai, Ruike An, Keyi Zhang, Xinyue Zhang, Hui An, Jitai Zhang, Yongyu Wang, Ming Li

**Affiliations:** 1 Department of Biochemistry and Molecular Biology, School of Basic Medical Sciences, Wenzhou Medical University, Wenzhou 325035, China; 17778912672@163.com (Q.Z.); 2662548575@wmu.edu.cn (H.C.); 17757728680@163.com (J.B.); ark17835207163@wmu.edu.cn (R.A.); zky15957651455@wmu.edu.cn (K.Z.); 2 Cardiac Regeneration Research Institute, School of Basic Medical Sciences, Wenzhou Medical University, Wenzhou 325035, China; qhdzxy@wmu.edu.cn (X.Z.); anhui@wmu.edu.cn (H.A.); jtaizhang@wmu.edu.cn (J.Z.); 3 Institute of Hypoxia Medicine, School of Basic Medical Sciences, Wenzhou Medical University, Wenzhou 325035, China; yongyuwang@wmu.edu.cn

In the original publication [1], there were some mistakes in Figures 5A and 7E as published. In Figure 5A, the top three panels in the left column incorrectly displayed duplicates of Panel 4 (bottom left panel) instead of the original data. In Figure 7E, the middle three panels (positions 2–4) in the top row erroneously showed duplicates of Panel 5 (rightmost panel). The corrected Figure 5 and Figure 7 appear below. The authors state that the scientific conclusions are unaffected. This correction was approved by the Academic Editor. The original publication has also been updated.

## Figures and Tables

**Figure 5:**
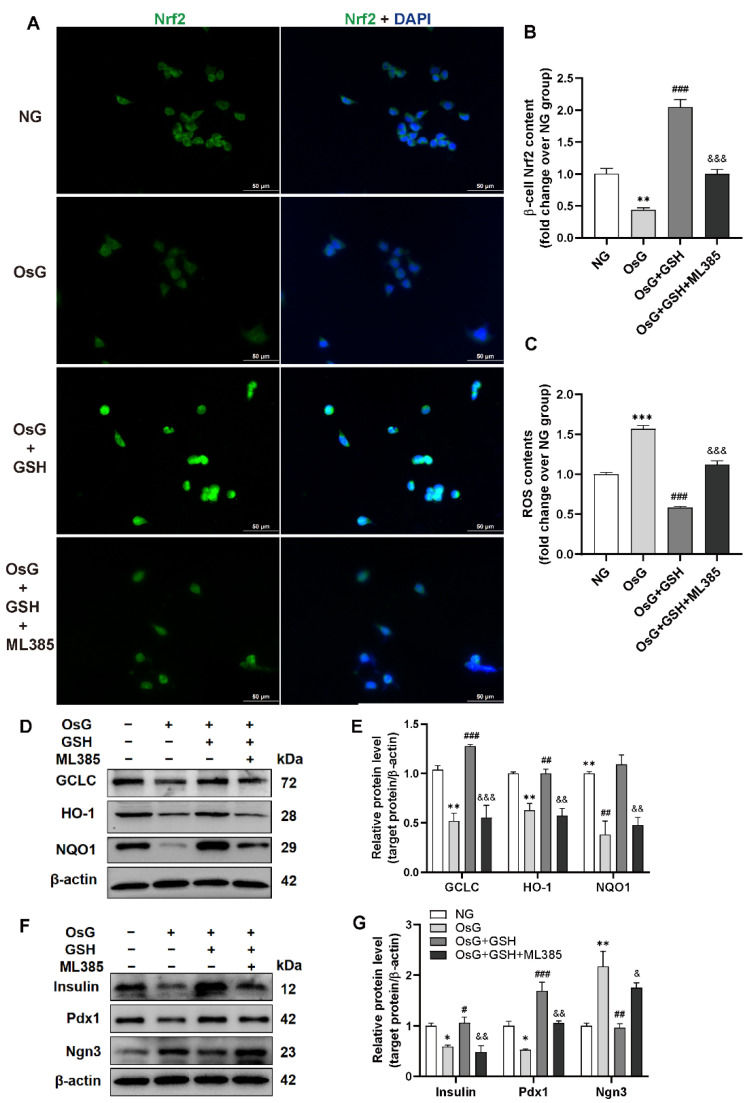
Blocking Nrf2 activity eliminates the protective effects of GSH in β-cells treated with OsG. RINm5F cells were cultured under oscillating glucose conditions with or without GSH for 4 days, followed by treatment with or without ML385 for an additional 2 days. (**A**) Immunofluorescence staining of Nrf2 (green) and DAPI (blue) was performed on RINm5F cells. Scale bars, 50 μm. (**B**) Quantification of Nrf2 staining (*n* = 4). (**C**) Measurement of ROS levels in RINm5F cells (*n* = 3). Representative western blots (**D**) and densitometry analysis of (**E**) GCLC, HO-1, and NQO1 protein levels (*n* = 3). Representative western blots (**F**) and densitometry analysis of (**G**) insulin, Pdx1, and Ngn3 protein levels (*n* = 3). Data shown are mean ± SEM. * *p* < 0.05, ** *p* < 0.01, *** *p* < 0.001 vs. NG group; ^#^ *p* < 0.05, ^##^ *p* < 0.01, ^###^ *p* < 0.001 vs. OsG group; ^&^ *p* < 0.05, ^&&^ *p* < 0.01, ^&&&^ *p* < 0.001 vs. OsG + GSH group.

**Figure 7:**
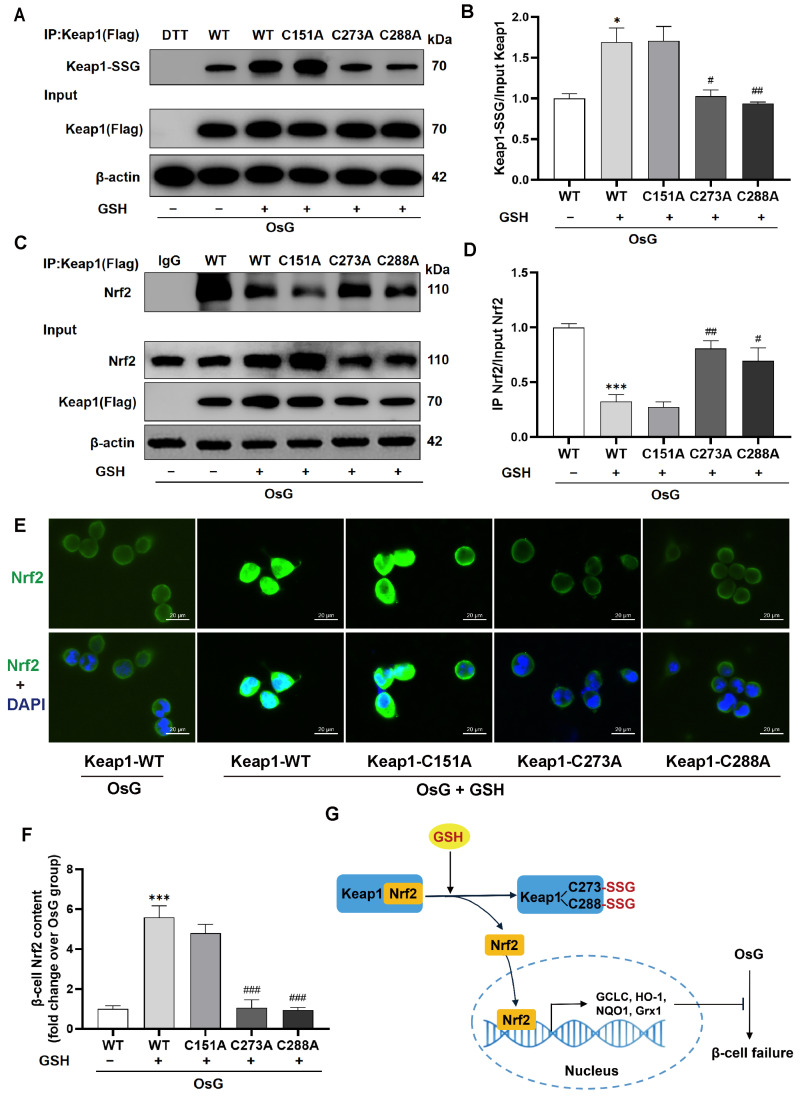
Inhibition of Keap1 S-glutathionylation by Cys273 and Cys288 mutations suppressed the activation of the Nrf2 pathway in GSH-treated β-cells under OsG conditions. RINm5F cells were cultured with oscillating glucose with or without GSH for 72 h prior to transfection, followed by plasmid transfection of Keap1-WT or mutated Keap1 at Cys151, Cys273, and Cys288 for 24 h and by OsG treatment in the presence or absence of GSH for an additional 72 h. (**A**) Cell lysates were immunoprecipitated using an anti-Flag antibody, and the immunoprecipitated proteins were analyzed by immunoblotting with anti-glutathione antibody (DTT served as a negative control). A portion of the total lysate was also analyzed using an anti-Flag antibody. (**B**) The level of S-glutathionylated Keap1 was quantified and normalized to the total Keap1 protein (*n* = 3). (**C**) Cell lysates were immunoprecipitated using an anti-Flag antibody and then immunoblotted with an anti-Nrf2 antibody. The total lysates were analyzed for the expression of Nrf2, Keap1 (Flag), and β-actin. (**D**) Quantitative analysis of Nrf2 immunoprecipitated by Keap1 and normalized to the total Nrf2 protein (*n* = 3). (**E**) Immunofluorescence staining was performed on cells using antibodies against Nrf2 (green) and DAPI (blue). Scale bars, 20 μm. (**F**) Quantification of Nrf2 staining (*n* = 10). Data shown are mean ± SEM. (**B**,**D**,**F**): * *p* < 0.05, *** *p* < 0.001 vs. Keap1-WT + OsG group; ^#^ *p* < 0.05, ^##^ *p* < 0.01, ^###^ *p* < 0.001 vs. Keap1-WT + OsG + GSH group. (**G**) The schematic diagram illustrates that GSH S-glutathionylates Keap1 at Cys273 and Cys288, leading to the dissociation of Nrf2 from Keap1. This, in turn, facilitates the translocation of Nrf2 into the nucleus, where it activates the transcription of antioxidant genes, thereby inhibiting OsG-induced β-cell failure.
